# Neutrophil-to-Lymphocyte and Platelet-to-Lymphocyte Ratio: Side by Side with Molecular Mutations in Patients with Non-Small Cell Lung Cancer—The INOLUNG Study

**DOI:** 10.3390/cancers16162903

**Published:** 2024-08-21

**Authors:** Corina Eugenia Budin, Iuliu Gabriel Cocuz, Liviu Sorin Enache, Ionuț Alexandru Rența, Cristian Cazacu, Dariana Elena Pătrîntașu, Mihai Olteanu, Ruxandra-Mioara Râjnoveanu, Edith Simona Ianoși, Armand Râjnoveanu, Ovidiu Simion Cotoi

**Affiliations:** 1 Pathophysiology Department, George Emil Palade University of Medicine, Pharmacy, Science, and Technology of Târgu Mureș, 540139 Târgu Mureș, Romania; corina.budin@umfst.ro (C.E.B.); iuliu.cocuz@umfst.ro (I.G.C.); ovidiu.cotoi@umfst.ro (O.S.C.); 2 Pneumology Department, Clinical County Hospital Mureș, 540136 Târgu Mureș, Romania; ionutrenta@gmail.com (I.A.R.); dpatrintasu@yahoo.com (D.E.P.); 3 Pathology Department, Clinical County Hospital Mureș, 540136 Târgu Mureș, Romania; 4 Emergency Clinical Hospital, 540136 Târgu Mureș, Romania; enachesliviu@yahoo.com; 5 “Dimitrie Cantemir” University, 540545 Târgu Mureș, Romania; 6 George Emil Palade University of Medicine, Pharmacy, Science, and Technology of Târgu Mureș, 540139 Târgu Mureș, Romania; cazacu.cristian99@yahoo.com; 7 Pneumology Department, University of Medicine and Pharmacy, 700115 Craiova, Romania; mihai.olteanu@umfcv.ro; 8 Victor Babeș Hospital, 200515 Craiova, Romania; 9 Palliative Medicine Department, Iuliu Hațieganu University of Medicine and Pharmacy Cluj Napoca, 400348 Cluj Napoca, Romania; 10 Leon Daniello Hospital, 400332 Cluj Napoca, Romania; 11 Pneumology Department, George Emil Palade University of Medicine, Pharmacy, Science, and Technology of Târgu Mureș, 540139 Târgu Mureș, Romania; edith.ianosi@umfst.ro; 12 Occupational Medicine Department, Iuliu Hatieganu University of Medicine and Pharmacy, 400348 Cluj-Napoca, Romania; armand.rajnoveanu@umfcluj.ro

**Keywords:** neutrophil-to-lymphocyte ratio (NLR), platelet-to-lymphocyte ratio (PLR), non-small cell lung cancer (NSCLC), molecular mutations, EGFR

## Abstract

**Simple Summary:**

This retrospective observational study recruited 380 patients, 115 with lung cancer and 265 in the control group. Both the neutrophil/lymphocyte ratio (NLR) and the platelet/lymphocyte ratio (PLR) were significantly higher in cancer patients than in the control group. The correlation of PLR with the probability of lung cancer differs between men and women. CRP values did not differ according to histological types of lung cancer (*p* = 0.242) and were not associated with the presence of mutations in EGFR (*p* = 0.679). CRP values were significantly lower in NSCLC patients with PD-L1 mutations than in those without PD-L1 mutations (22.7 [IQR: 10.4;40.3] vs. 76.7 [IQR: 32.6;140] mg/L, *p* = 0).

**Abstract:**

*Background and objective:* Analysis of inflammatory biomarkers, along with the neutrophil/lymphocyte ratio (NLR) or platelet/lymphocyte ratio (PLR), supports the connection between inflammation and carcinogenesis. *Methods:* We conducted a retrospective observational study at the Clinical County Hospital Mureș involving patients with lung cancer. The parameters analyzed included histopathological type (NSCLC: squamous cell carcinoma or adenocarcinoma; SCLC), molecular mutations (EGFR, ALK, PD-L1), parameters from the complete blood count, inflammatory parameters, and associated comorbidities. *Results:* A total of 380 patients were included: 115 patients in the cancer group and 265 patients in the control group. Among patients in the lung cancer group, 88 were diagnosed with NSCLC (44 adenocarcinomas, 44 squamous cell carcinomas) and 27 with SCLC. Both NLR and PLR were significantly higher in cancer patients than in the control group (5.30 versus 2.60, *p* < 0.001; 217 versus 136, *p* < 0.001, respectively). NLR and PLR differ between men and women (*p* = 0.005 and *p* = 0.056, respectively). C-reactive protein was not correlated with either NLR (*p*-value: 0.0669) or PLR (*p*-value: 0.6733) in lung cancer patients. *Conclusions:* The NLR and PLR values may serve as new predictive biomarkers for the diagnosis of disease in patients with lung cancer, especially those with NSCLC.

## 1. Introduction

Lung cancer, which has the highest mortality rate among neoplasms, is often only diagnosed in advanced stages of the disease. The main histopathological category is non-small cell lung cancer (NSCLC; 85% of cases), which, in turn, includes adenocarcinomas (AdCs), squamous cell carcinomas (SCCs), and large cell carcinomas (LCCs) [1]. Common factors such as smoking, comorbidities, and histopathological diagnosis (histological type, molecular mutations, positive tumor immunostaining) correlate with the aggressiveness of the disease, prognosis, survival, and treatment response [2]. The most frequent mutant genes are p53, Kirsten rat sarcoma viral oncogene (KRAS), epidermal growth factor receptor (EGFR), mesenchymal–epithelial transition factor (MET) [3], and anaplastic lymphoma kinase (ALK) [1,2]. Systemic inflammation amplification correlates with an unfavorable prognosis in neoplastic patients. C-reactive protein (CRP) can directly interact with components of the extracellular matrix (fibroblasts) [4], which are constituents of the tumor stroma [5,6,7,8].

An elevated value of the neutrophil-lymphocyte ratio (NLR) is associated with increased peritumoral macrophage infiltration and increased values of other biomarkers, such as IL1, IL7, IL8, or IL17 [4]. The systemic inflammatory response is associated with changes in neutrophil values (neutrophilia) and relative lymphocytopenia [9]. Hematological tests are routinely performed for cancer patients in a variety of clinical scenarios and represent easily measurable objective parameters capable of expressing the severity of the systemic inflammatory response in neoplastic patients [10]. NLR may represent the balance between pro-tumoral inflammatory status and anti-tumoral immune response [10]. Increased inflammatory biomarkers have been shown to be associated with an unfavorable prognosis in both neoplastic diseases and infectious diseases, such as pulmonary tuberculosis, bronchiectasis [11], and SARS-CoV-2 infection [12]. The connection between inflammation and carcinogenesis supports the fact that determining inflammatory biomarkers, along with the lymphocyte/neutrophil ratio or platelet/lymphocyte ratio, could represent new directions for estimating the evolution and prognosis of neoplastic diseases [13].

## 2. Materials and Methods

A retrospective study of patients with lung cancer was conducted at the Clinical County Hospital Mureș, Târgu Mureș. The patients were hospitalized between 1 February 2023 and 31 December 2023.

This human study was performed in accordance with the Declaration of Helsinki and it was approved by the ethics committee of Clinical County Hospital Mureș (approval: 6089/28.05.2024). All adult participants provided written informed consent to participate in this study.

Patients with a histopathologically confirmed diagnosis of lung cancer following endobronchial biopsy during the period of 1 February to 31 December 2023 were included in the lung cancer patient group. The control group consisted of patients admitted to the Pulmonology Clinic of the same hospital during the same period (1 February to 31 December 2023), with non-neoplastic diagnoses. The hospital admission period was similar in order to have statistical compatibility between the two analyzed groups.

The analysis of the data presented in this study is part of larger ongoing research and constitutes the INOLUNG (innovation, lung) Study.

Inclusion criteria for patients in the lung cancer group: (1) patients with histopathological confirmation of lung cancer; (2) patients over 18 years of age; (3) patients for whom the analyzed variables were available. Exclusion criteria for the lung cancer patient group: (1) patients with clinical or imaging suspicion of lung cancer but without histopathological confirmation; (2) patients under 18 years of age; (3) patients for whom the analyzed variables were not available.

Inclusion criterion for patients in the control group: patients continuously hospitalized in the Pulmonology Clinic of the Clinical County Hospital Mureș during the period of 1 February to 31 December 2023. Exclusion criteria for patients in the control group: (1) patients hospitalized in the Pulmonology Clinic at a time other than 1 February to 31 December 2023; (2) patients hospitalized with suspicion of lung cancer; (3) patients hospitalized with a diagnosis of pulmonary tuberculosis or SARS-CoV-2 infection.

The analyzed parameters included histopathological type (NSCLC: squamous cell carcinoma or adenocarcinoma; SCLC), molecular mutations (EGFR, ALK, PD-L1), parameters from the complete blood count (hemoglobin level, leukocyte count, platelet count, NLR, PLR, lymphocyte count), inflammatory markers (C-reactive protein), associated comorbidities, and disease stage.

### Statistical Analysis

Raw data were collected retrospectively from medical records and stored in Microsoft Excel (Version 16.78.3 (231102801), 2019) spreadsheets. Statistical analysis was performed in the R statistical environment (version 4.3.3, released on 29 February 2024). Continuous variables were checked for Gaussian distribution using the Shapiro–Wilk test. Comparisons between groups regarding numerical variables were performed using the Wilcoxon or Kruskal–Wallis rank sum tests. Categorical variables were compared using Fisher’s Exact Test for count data. Prior to fitting multiple logistic regression models, we performed an analysis of missing data followed by multiple imputation via chained equations. Throughout the study, a statistical significance threshold of 0.05 was used.

## 3. Results

In concordance with the inclusion and exclusion criteria, we included data from 380 patients: 115 in the cancer group and 265 in the control group. The characteristics of the participants are summarized in Table 1.

Among the patients in the lung cancer group, 88 were diagnosed with NSCLC (44 adenocarcinomas, 44 squamous cell carcinomas) and 27 with SCLC. The presence of mutations was assessed for EGFR, ALK, and PD-L1 genes in 68 patients with NSCLC. Molecular analysis for PD-L1 failed in one patient due to insufficient sample quantity. EGFR mutations were detected in 7 patients (10.3% of the 68 tested), whereas ALK and PD-L1 mutations were identified in 1 (1.5%) and 25 (37.3%) patients, respectively. EGFR mutations involved exon 18 in one case (1.5%), exon 19 in two cases (2.9%), exon 20 in one case (1.5%), and exon 21 in three cases (4.4%).

We did not observe differences in age or gender distribution between patients with and without EGFR mutations. However, regarding the histological type of the tumor, adenocarcinomas were more frequent than squamous cell carcinomas in patients with EGFR mutations (85.7% vs. 14.3%, *p* = 0.046, Table 2).

We observed no association of age, gender, or histological subtype with the presence of mutations in PD-L1 in patients with NSCLC. PD-L1 mutation status did not seem to influence NLR or PLR values in these patients either (Table 3).

Disease stage at the time of diagnosis was not associated with either EGFR or PD-L1 mutations (*p* = 1 and 0.860, respectively). Mortality, assessed from medical records 14 months after the beginning of the study, was not influenced by PD-L1 mutations, but tended to be associated with EGFR mutations, without reaching the statistical significance level (*p* = 0.085).

Smoking is a known risk factor for lung cancer. In our study, the median number of packs/year in cancer patients was four times higher than in control patients (*p* < 0.001, Table 1). Also, the prevalence of COPD in lung cancer patients was higher than in the control patients (*p* < 0.001). When examining the association of EGFR mutations with smoking, we observed that cancer patients with EGFR mutations had a significantly lower tobacco cigarette consumption than those with wild-type EGFR (median of 5 [interquartile range, IQR: 0; 17.5] vs. 40 [IQR: 30; 50] packs/year, *p* = 0.002), suggesting that EGFR mutagenesis occurs independently of smoking or that smoking promotes carcinogenesis through mechanisms that do not involve EGFR mutations. The number of cigarette packs-year was not associated with PD-L1 mutations (*p* = 0.542).

Regarding other comorbidities, although the diagnosis of COPD was most frequently associated with lung cancer, as previously mentioned, when analyzing the two groups of patients we observed that the association of cardiovascular pathologies as well as renal pathologies was relatively similar in the group of cancer patients and in the control group, while the association of diabetes mellitus was higher in the control group versus the group of cancer patients (n = 26; 26% versus n = 20.18%) (Table 1). Asthma appears to be more common in patients with EGFR mutations than in those without mutations (50% vs. 6.8%, *p* = 0.014), but this result is recommended to be interpreted with caution given the small number of cases.

Nutrition status was poorer in lung cancer patients compared with that of the control group, a condition reflected by the lower BMI in the former group (*p* < 0.001). Hemoglobin levels were also lower (*p* < 0.001) in the cancer group, despite the higher proportion of males than females in this group compared with the control group (Table 1). However, neither BMI nor hemoglobin values were associated with the tumor histological type (*p* = 0.934 and *p* = 0.669).

**Figure 1:**
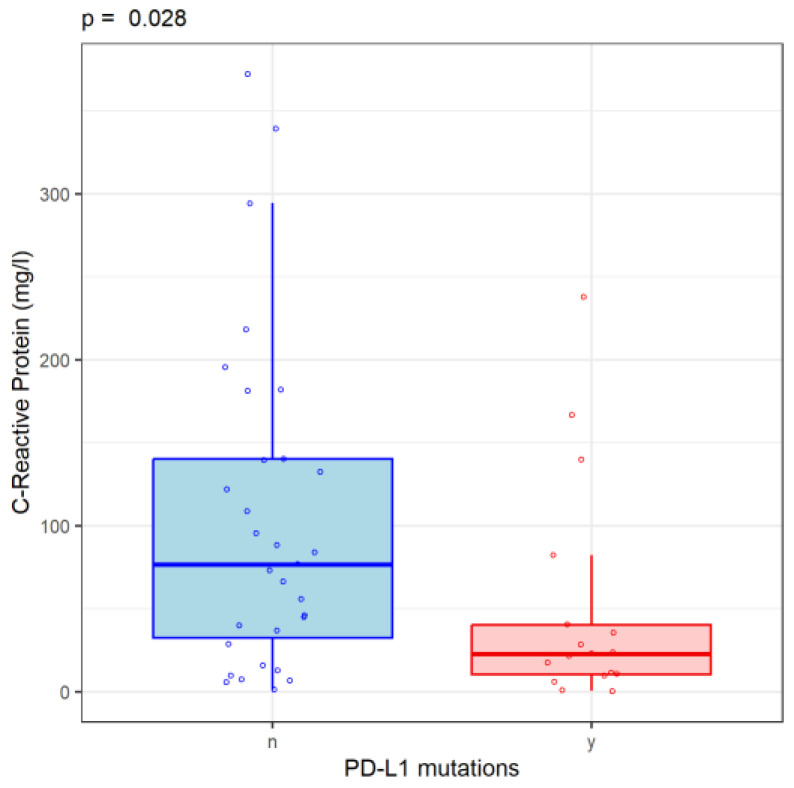
CRP value in PD-L1 mutation patients. y = presence of PD-L1 mutation; n = no PD-L1 mutation.

Serum C-reactive protein (CRP) was assessed in cancer patients as a non-specific marker of inflammation. CRP values did not differ according to the histological type of lung cancer (*p* = 0.242) and were not associated with the presence of mutations in EGFR (*p* = 0.679). CRP values were significantly lower in NSCLC patients with PD-L1 mutations than in those without PD-L1 mutations (22.7 [IQR: 10.4; 40.3] vs. 76.7 [IQR: 32.6; 140] mg/L, *p* = 0.029) (Figure 1).

**Figure 2:**
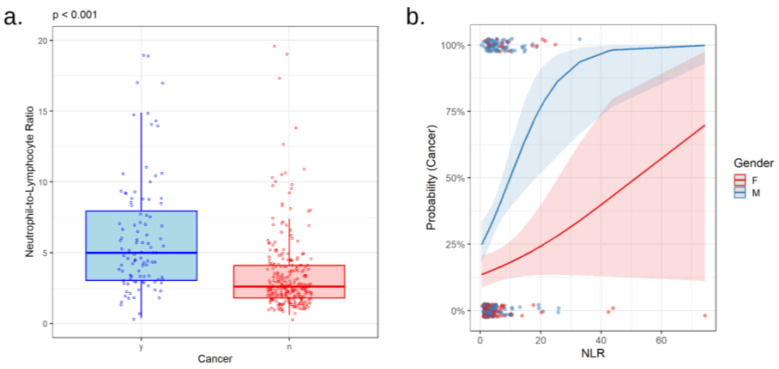
Neutrophil-to-lymphocyte ratio (NLR) in relation to lung cancer (**a**). Comparison of NLR values in patients with (y) or without (n) lung cancer (**b**). Correlation between NLR values with the probability of lung cancer in males (M) and females (F).

**Figure 3:**
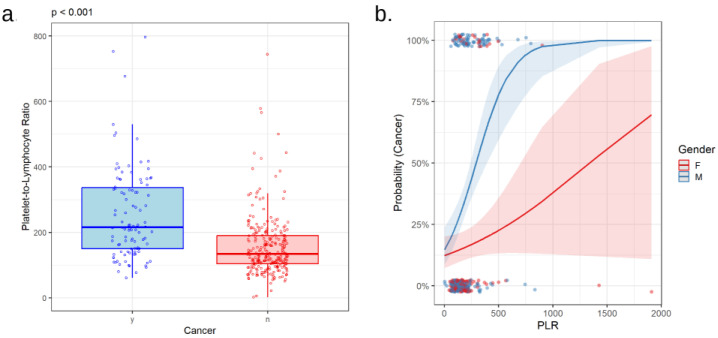
Platelet-to-lymphocyte ratio (PLR) in relation to lung cancer (**a**). Comparison of PLR values in patients with (y) or without (n) lung cancer (**b**). Correlation between PLR values with the probability of lung cancer in males (M) and females (F).

Complete blood counts revealed higher numbers of neutrophils and platelets, as well as lower numbers of lymphocytes in patients with lung cancer compared to those in the control group (*p* < 0.001, *p* < 0.001, and *p* = 0.002, respectively; Table 1). Accordingly, both NLR and PLR were higher in cancer patients (*p* < 0.001 for both, Figure 2a and Figure 3a). Neither NLR nor PLR varied with tumor histological type. In patients with adenocarcinoma, squamous cell carcinoma, and SCLC, NLR values were 5.30 [3.27; 8.47], 5.30 [3.50; 8.55], and 4.45 [2.58; 7.30], respectively (*p* = 0.580), whereas PLR values were 218 [150; 366], 215 [156; 326], and 208 [130; 324], respectively (*p* = 0.448). NLR and PLR were not associated with the presence of mutations in EGFR (*p* = 0.664 and *p* = 0.645) or PD-L1 (*p* = 0.233 and *p* = 0.316) genes.

Interestingly, the correlation of PLR with the probability of lung cancer differs between men and women (*p* = 0.005 for the interaction between the terms PLR and gender in logistic regression for the presence of cancer, Figure 3b). The same trend was observed for the interplay of NLR and gender in association with cancer, although at the limit of statistical significance in our group (*p* = 0.056, Figure 2b).

**Figure 4:**
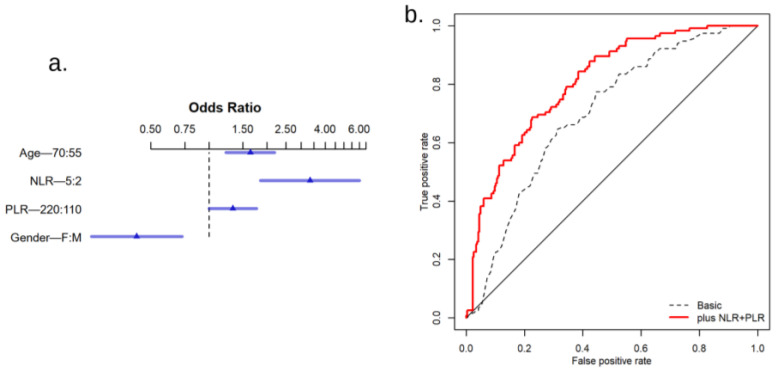
Predictive model for the presence of lung cancer in patients with pulmonary disease (**a**). Odds ratios (triangles) and their 95% confidence intervals (horizontal bars) for the components of the predictive model: Age, Gender, NLR, and PLR (**b**). Receiver operating characteristic (ROC) curves for a basic model (based on Age and Gender only) and the complete model (based on Age, Gender, NLR, and PLR) based on multiple logistic regression for the prediction of the presence of lung cancer. The closer the curve to the top-left corner of the chart, the more accurate the distinction between a patient with and a patient without cancer.

Given the differences in age and gender distribution between our cancer and control groups, we sought to verify whether these variables may be confounders for the differences observed for NLR and PLR in these groups. However, when adjusting for gender and age in multiple logistic regression, NLR and PLR retained their significance as predictive factors for the diagnosis of lung cancer (*p* < 0.001 and *p* = 0.048, respectively; Figure 4a). In addition, a logistic predictive model based on age, gender, NLR, and PLR showed superior discriminative power between cancer and control patients, compared with a predictive model including only age and gender (AUC of 0.777 [0.772–0.782] vs. 0.700 [0.694–0.706], Figure 4b).

## 4. Discussion

NLR and PLR, two novel inflammatory biomarkers, are part of the predictive profile of cancer patients [14]. Some studies in the literature used these parameters to evaluate the response of cancer patients to oncological treatment, especially immunotherapy [15,16]. Beyond this aspect, correlated with targeted oncological therapy, few studies have addressed the predictive potential diagnostic model of these new inflammatory biomarkers [17,18].

The NLR and PLR values in patients with lung cancer have been particularly used in patients with NSCLC. Although the objective of our study aimed to analyze these parameters in NSCLC patients, in the cohort of lung cancer patients (n = 115), 27 patients with SCLC were included. In a systematic review published in 2021 by Larsen et al. [19], the correlation between an increased NLR value in SCLC patients and increased mortality was presented. In our study, the NLR value was lower in patients with SCLC than in those with NSCLC, but mortality could not be fully analyzed as it is a parameter that will need to be evaluated in subsequent follow-up research.

In other research, NLR and PLR have been used in clinical practice to evaluate postoperative prognostic potential [20], but the cancer patients included in our study were in advanced stages of the disease, where surgical intervention is no longer recommended, so this parameter was not monitored.

The coexistence of a diagnosis of lung cancer and COPD increased the NLR and PLR values [21], a finding also present in our study.

Research conducted by Dan Pu et al. highlighted that, in NSCLC patients with PD-L1 mutation, an increased NLR value is associated with a poor prognosis and serves as an individual predictive factor [16,22]. The cutoff values used were 5 for NLR and 200 for PLR [22]. In our study, the average NLR value was positioned below this cutoff value (4.50 [2.85; 5.92]), and the same result was found for PLR (198 [141; 304]). The limitation of our study, i.e., not analyzing the differentiated group of patients with PD-L1 mutation, stems from the restricted number of patients; thus, the data would not have presented statistical validity.

As early as 2014, the study led by Kim et al. claimed that smoking over 30 cigarette packs-year is an independent negative predictive factor of targeted treatment with EGFR-TKI [23]. Although our research did not include assessing the impact of immunotherapy, when examining the association of EGFR mutations with smoking, we observed that cancer patients with EGFR mutations had a significantly lower tobacco cigarette consumption than those with wild-type EGFR. On the other hand, we did not observe differences in terms of age nor gender distribution between patients with or without EGFR mutations.

Analyzing other data from the literature, we found the same cutoff value of 5 for NLR [24], with values above 5 being associated with a low performance index and poor survival [14]. For PLR, the cutoff value of approximately 200 also correlates with survival and clinical status; values above 200 in PLR are associated with a poor prognosis [2,6]. In our study, the average NLR value in the group of cancer patients was 5.3, and the average PLR value was 217. These average values are above the limit found in the literature, which could be explained by the fact that most patients were in advanced stages of the disease at the time of diagnosis. The comparative analysis of these parameters versus the control group confirms the validity of using NLR and PLR as inflammatory biomarkers, even though the data are insufficient to support their roles in a predictive model.

The assessment of these two parameters, NLR and PLR, was made, both in our study and in the literature, with the aim of being used as potential diagnostic factors for lung cancer patients [10], especially those with NSCLC [24,25]. The utility of these two easily accessible ratios from routine patient analyses has been used not only for lung cancer but also for hepatocellular carcinoma, gastric cancer, and prostate cancer [13,26]. Along with these two parameters, other accessible patient characteristics such as age, gender, body mass index, inflammatory profile, and serological, immunohistochemical, or molecular determinations can also be used [27,28]. Regarding gender, a study conducted on Chinese patients published in 2021 revealed NLR in males was significantly higher than in females, but this study only included healthy adults [29]. Also, in our study the probability of lung cancer differed between men and women, even if our population included lung cancer patients. The cumulative analysis of these parameters could contribute to the creation of a predictive model of the evolution of lung cancer patients. In our patient cohort, these parameters were analyzed separately, trying to establish whether they could be contributing factors to this predictive model.

This study constitutes a pilot project which involved creating a local database; this aspect is a limitation of the study, as it was carried out at a single site. By expanding this cohort, a predictive model with statistical significance could be developed to guide therapeutic management. Another hypothesis would be whether these two parameters could be used as adjunct factors in the diagnostic stage of lung cancer, with their increased value necessitating a more detailed screening of patients.

One of the limitations of our study includes the fact that the NLR value was assessed at the time of oncological diagnosis when the biopsy sample was taken and the NLR value was not assessed pre- and post-oncological treatment or pre- and post-immunotherapy. Another limitation is that we only used Caucasian individuals, and the value of inflammatory biomarkers may vary.

In the literature, there are no commonly accepted values for NLR and PLR. As presented, the most commonly used cutoff values are 5 and 200, respectively [30]. Since there are no universally accepted values, we did not use a cutoff value, but calculated the average value for the two analyzed groups. To facilitate the assessment of these biomarkers, a universally accepted standard value should be established [10,13]. This would represent clinical utility both at the time of diagnosis as a predictive factor of evolution and throughout therapy to assess response to treatment, especially targeted immunotherapy [31].

## 5. Conclusions

Along with other biometric, clinical, imaging, and serological tests, the value of NLR and PLR can constitute new predictive biomarkers of diagnosis and disease progression in lung cancer patients, especially those with NSCLC.

## Figures and Tables

**Table 1:** Summary of characteristics ^a^ of the patients included in the study.

	[ALL]	Cancer	Control	*p*-Value	N ^b^
	N = 380	N = 115	N = 265		
Age	64.0 [54.0; 71.0]	68.0 [62.5; 74.0]	61.0 [51.0; 70.0]	<0.001	380
Gender:				<0.001	380
F	153 (40.3%)	28 (24.3%)	125 (47.2%)		
M	227 (59.7%)	87 (75.7%)	140 (52.8%)		
Lymphocytes, ×10^3^/µL	1.73 [1.24; 2.33]	1.47 [1.07; 2.09]	1.80 [1.33; 2.38]	0.002	368
Neutrophils, ×10^3^/µL	5.51 [4.11; 7.92]	7.92 [5.65; 10.4]	4.86 [3.85; 6.61]	<0.001	369
Platelets, ×10^3^/µL	270 [216; 336]	336 [269; 402]	252 [203; 308]	<0.001	368
Hemoglobin, g/dL	13.4 [12.2; 14.5]	12.8 [11.2; 14.0]	13.5 [12.6; 14.8]	<0.001	360
Smoking, packs/year	20.0 [0.00; 40.0]	40.0 [20.0; 50.0]	10.0 [0.00; 30.0]	<0.001	337
COPD:				<0.001	378
n	194 (51.3%)	31 (27.4%)	163 (61.5%)		
y	184 (48.7%)	82 (72.6%)	102 (38.5%)		
Cardiovascular pathologies				0.1	2212
n	58 (27.4%)	31 (27.7%)	27 (27.0%)		
y	154 (72.6%)	81 (72.3%)	73 (73.0%)		
Diabetes mellitus				0.217	2211
n	165 (78.2%)	91 (82.0%)	74 (74.0%)		
y	46 (21.8%)	20 (18.0%)	26 (26.0%)		
Renal pathologies				0.1	2209
n	194 (92.8%)	101 (92.7%)	93 (93.0%)		
y	15 (7.18%)	8 (7.34%)	7 (7.00%)		
BMI, kg/m^2^	27.0 [23.4; 30.7]	24.6 [21.4; 27.3]	29.3 [26.0; 33.1]	<0.001	144
NLR	3.00 [2.00; 5.30]	5.30 [3.15; 8.35]	2.60 [1.80; 4.30]	<0.001	368
PLR	153 [109; 218]	217 [150; 342]	136 [105; 192]	<0.001	367

^a^ Values are presented as numbers (percentages) for categorical variables and medians [interquartile range] for numerical variables. ^b^ Number of observations for a given variable. COPD = chronic obstructive pulmonary disease.

**Table 2:** Characteristics ^a^ of NSCLC patients in relation to the presence of EGFR mutations.

	[ALL]	EGFR: Wild-Type	EGFR: Mutated	*p*-Value	N ^b^
	N = 68	N = 61	N = 7		
Age	68.5 [63.0; 73.2]	68.0 [63.0; 73.0]	71.0 [60.0; 73.5]	0.76	68
Gender:				0.18	68
F	15 (22.1%)	12 (19.7%)	3 (42.9%)		
M	53 (77.9%)	49 (80.3%)	4 (57.1%)		
Histological type				0.05	68
adenocarcinoma	32 (47.1%)	26 (42.6%)	6 (85.7%)		
squamous cell carcinoma	36 (52.9%)	35 (57.4%)	1 (14.3%)		
Stage:				1	53
2	1 (1.89%)	1 (2.00%)	0 (0.00%)		
3	28 (52.8%)	26 (52.0%)	2 (66.7%)		
4	24 (45.3%)	23 (46.0%)	1 (33.3%)		
Lymphocytes, ×10^3^/µL	1.51 [1.14; 2.02]	1.49 [1.12; 2.06]	1.99 [1.41; 2.00]	0.54	61
Neutrophils, ×10^3^/µL	7.72 [5.90; 10.4]	7.70 [5.89; 9.97]	7.92 [6.45; 10.5]	0.79	61
Platelets, ×10^3^/µL	330 [269; 426]	334 [273; 428]	281 [263; 337]	0.58	61
C-reactive protein, mg/L	51.0 [17.2; 140]	61.2 [20.8; 140]	25.4 [10.2; 123]	0.65	48
Hemoglobin, g/dL	12.8 [11.2; 13.9]	13.0 [11.3; 14.0]	11.8 [10.8; 12.3]	0.38	58
Smoking, packs/year	35.0 [24.0; 50.0]	40.0 [30.0; 50.0]	5.00 [0.00; 17.5]	0	65
COPD:				0	67
no	21 (31.3%)	15 (24.6%)	6 (100%)		
yes	46 (68.7%)	46 (75.4%)	0 (0.00%)		
BMI, kg/m^2^	24.5 [19.9; 27.3]	24.5 [20.3; 26.8]	21.7 [17.9; 25.5]	0.86	35
NLR	5.00 [3.20; 8.00]	5.15 [3.25; 8.03]	3.30 [3.20; 5.30]	0.66	61
PLR	217 [150; 333]	218 [151; 334]	187 [141; 321]	0.64	61

^a^ Values are presented as numbers (percentages) for categorical variables and medians [interquartile range] for numerical variables. ^b^ Number of observations for a given variable. COPD = chronic obstructive pulmonary disease.

**Table 3:** Characteristics ^a^ of NSCLC patients in relation to the presence of PD-L1 mutations.

	[ALL]	PD-L1: Wild-Type	PD-L1: Mutated	*p*-Value	N ^b^
	N = 67	N = 42	N = 25		
Age	69.0 [63.0; 74.0]	69.0 [63.0; 71.8]	68.0 [64.0; 74.0]	0.68	67
Gender:				0.16	67
F	14 (20.9%)	6 (14.3%)	8 (32.0%)		
M	53 (79.1%)	36 (85.7%)	17 (68.0%)		
Histological type				0.64	67
adenocarcinoma	31 (46.3%)	18 (42.9%)	13 (52.0%)		
squamous cell carcinoma	36 (53.7%)	24 (57.1%)	12 (48.0%)		
Stage:				0.86	52
2	1 (1.92%)	1 (3.03%)	0 (0.00%)		
3	28 (53.8%)	17 (51.5%)	11 (57.9%)		
4	23 (44.2%)	15 (45.5%)	8 (42.1%)		
Lymphocytes, ×10^3^/µL	1.49 [1.08; 2.06]	1.52 [1.12; 1.99]	1.40 [1.07; 2.39]	0.87	60
Neutrophils, ×10^3^/µL	7.78 [5.89; 10.4]	8.15 [6.80; 10.5]	6.28 [5.24; 9.46]	0.09	60
Platelets, ×10^3^/µL	338 [276; 428]	60 [279; 442]	322 [256; 361]	0.26	60
C-reactive protein, mg/L	45.3 [15.3; 134]	76.7 [32.6; 140]	22.7 [10.4; 40.3]	0.03	48
Hemoglobin, g/dL	12.6 [11.2; 13.7]	12.2 [10.8; 14.0]	13.1 [11.9; 13.7]	0.28	57
Smoking, packs/year	39.0 [24.8; 50.0]	38.0 [24.5; 47.5]	40.0 [30.0; 60.0]	0.54	64
COPD:				1	66
no	21 (31.8%)	13 (31.7%)	8 (32.0%)		
yes	45 (68.2%)	28 (68.3%)	17 (68.0%)		
BMI, kg/m^2^	24.5 [19.9; 26.6]	23.6 [19.5; 25.3]	25.8 [24.6; 28.0]	0.08	35
NLR	5.15 [3.18; 8.15]	5.30 [3.45; 8.95]	4.50 [2.85; 5.92]	0.23	60
PLR	220 [151; 340]	227 [159; 362]	198 [141; 304]	0.31	60

^a^ Values are presented as numbers (percentages) for categorical variables and medians [interquartile range] for numerical variables. ^b^ Number of observations for a given variable. COPD = chronic obstructive pulmonary disease.

## Data Availability

No new data were created or analyzed in this study. Data sharing is not applicable to this article.
